# Effects of Combining Meditation Techniques on Short-Term Memory, Attention, and Affect in Healthy College Students

**DOI:** 10.3389/fpsyg.2021.607573

**Published:** 2021-03-05

**Authors:** Samani Unnata Pragya, Neelam D. Mehta, Bassam Abomoelak, Parvin Uddin, Pushya Veeramachaneni, Naina Mehta, Stephanie Moore, Melissa Jean-Francois, Stephanie Garcia, Samani Chaitanya Pragya, Devendra I. Mehta

**Affiliations:** ^1^Department of Religions and Philosophies, University of London, London, United Kingdom; ^2^Sidney Kimmel Medical College, Thomas Jefferson University, Philadelphia, PA, United States; ^3^Gastrointestinal Translational Laboratory, Arnold Palmer Hospital for Children, Orlando, FL, United States; ^4^College of Arts, Sciences and Education, Florida International University, Miami, FL, United States; ^5^College of Law, Florida International University, Miami, FL, United States; ^6^Department of Biostatistics, Robert Stempel College of Public Health and Social Work, Florida International University, Miami, FL, United States

**Keywords:** meditation, short-term memory, attention, affect, college students

## Abstract

Meditation refers to a family of self-regulation practices that focuses on training attention and awareness to foster psycho-emotional well-being and to develop specific capacities such as calmness, clarity, and concentration. We report a prospective convenience-controlled study in which we analyzed the effect of two components of *Preksha Dhyāna* – buzzing bee sound meditation (*Mahapran dhvani*) and color meditation (*leśyā dhyāna*) on healthy college students. *Mahapran* and *leśya dhyāna* are two *Preksha Dhyāna* practices that are based on sound and green color, respectively. The study population represents a suitable target as college students experience different stress factors during the school year. This study measures the individual and combined effects of two techniques (one focusing on sound and one focusing on color), on short-term memory, attention, and affect, in novice meditators. We used a battery of cognitive, performance, and compared results with baseline and control values. We found improved cognition, especially attention, short-term memory, and affect in terms of positivity and reduced negativity. Overall, the two techniques produced variable benefits and subjects showed improved scores over baseline for short-term memory, cognitive function, and overall wellbeing. Further studies are required to understand underlying mechanisms for the observed differences between the two techniques and to elucidate mechanisms underlying the more pronounced and global benefits observed with the combined techniques. These results underscore a need to examine individual components of meditation practices in order to individualize treatment approaches for attention disorders in young adults.

**Clinical Trail Registration:**ClinicalTrials.gov Identifier: NCT03779269.

## Introduction

Meditation is defined as a group of self-regulation practices used to focus an individual’s attention in order to achieve increased voluntary control over various psycho-emotional and physiologic states (Davis and Hayes, 2011). It is an ancient practice originating around 1,500 BCE in India and is increasingly used as a modality to treat physical and psycho-emotional disorders, including anxiety (Mehta et al., 2011; Hoge et al., 2013; Satsangi and Brugnoli, 2018), attention deficit/hyperactivity disorder (ADHD; Poissant et al., 2019), chronic pain (Reiner et al., 2013; Banth and Ardebil, 2015; Ball et al., 2017; Majeed et al., 2018), depression (Tolahunase et al., 2018; Carpena et al., 2019), irritable bowel syndrome (Gaylord et al., 2011), and post-traumatic stress disorder (PTSD; Gallegos et al., 2017). Meditation also has been found to increase focus and attention among adolescents and young adults (Jha et al., 2007; Black et al., 2009; Serwacki and Cook-Cottone, 2012). Recently, data showed that mindfulness is inversely associated with depression and stress among medical students (Alzahrani et al., 2020).

Many meditation practices involve a multistep process using two or more techniques to focus the mind on a specific stimulus (Hu et al., 2011).

Some studies have compared different whole-systems meditation practices in experienced meditators. A comparison of the effects of mindfulness-based meditation, a form of meditation with roots from Buddhism, and focused meditation (Dunn et al., 1999; Sevinc et al., 2018) showed each practice produced different electroencephalography (EEG) patterns in meditation groups compared with controls. A study of highly-trained Buddhist monks showed no observable changes resulting from compassion meditation (techniques to focus on cultivating deep concern for others; Hofmann et al., 2011), but a significant increase in the duration of perceptual dominance resulting from meditation focused on one point of the body (such as the navel).

Many studies have compared the effects of different meditation practices in experienced meditators (Amihai and Kozhevnikov, 2014; Sevinc et al., 2018), but few have assessed the effects of individual techniques or the impact of those techniques in novice meditators. Hirshberg et al. (2018) reported divergent effects of brief meditation in response to an acute stressor in a randomized controlled trial of 156 undergraduate students. The participants were novice to mindfulness, and they were allocated to one of four brief practices including breath awareness, loving-kindness, gratitude, or to an attention control condition. The study revealed an increased positivity although effects differences were noticed, especially after a stressor (Hirshberg et al., 2018). Our goal was to study the effects in novice meditators of two individual techniques used in the meditative practice, *preksha dhyana*. Furthermore, our study planned to assess the impact when the techniques were combined. In addition, we wanted to embed these techniques in their normal college time rather than vacation or summer camps to assess the impact at times of highest need. Although techniques used in *preksha dhyana* are similar to those used in mindfulness-based meditation, *preksha dhyana* also uses two techniques that focus on sound and on imagery (color). Enhanced understanding of these individual techniques could enable specific combinations to be used for personalized therapeutic approaches.

## Materials and Methods

### Ethical Considerations

The proposal was approved by the Florida International University (FIU) ethics, and the IRB committees. All participants (*n* = 142) signed consent forms in order to be enrolled in the study. All participants were given ID numbers according to the IRB rules (IRB-17-0108-CR02). Students received an IRB-approved small stipend for participating. The study was registered in Clinicaltrials.gov as NCT03779269. Exclusion criteria included previous use of any form of meditation and attendance of less than 80% of the intervention or control sessions.

### Techniques Studied

*Preksha Dhyana* is based on wisdom culture of ancient Jain ascetics that aims for pure consciousness. Meditation (Dhyana) and relaxation are core to this practice and have recently been compiled and formulated as a complete system by the renowned Jain ascetic, Acharya Mahapragya. The PI is a pupil of the former Acharya and an expert exponent of the system (Mahapragya, 1995). *Mahapran* is a sound-based technique (the English translation of *naad* is “sound”) created using either inner bodily sounds, ambient sounds, or basic universal sounds (Suriji Maharaja, 1997). We used a basic universal sound of bee-like buzzing (achieved using several deep breaths and exhaling with closed lips for several seconds at a time while focusing on the vibrations) to achieve intense concentration. We also studied a technique that includes focusing on an imaginary color. Participants concentrated on a visualized green point on the forehead (as used in Jain Yoga; Mahapragya, 1995), since bright green is associated with attention arousal (Valdez and Mehrabian, 1994) and with the heart chakra (one of several psychic energy centers in the body regarded as the ultimate focal point linking emotional, physical, and spiritual energies; Tulsi, 1994). We assessed both techniques individually and combined, using a battery of cognitive, performance, and compared results with baseline and control values. The study items were selected based on theoretical considerations that cognition, memory, and affect are important in overall assessment of any changes from such interventions. The selected scales were based on previous experience as well as validation with appropriate normative values for our age group.

### Procedure

The study was completed over the course of six school semesters (one semester = 3 calendar months) with new participants each semester. From the outset of the study, the design was to assess the two techniques separately and when combined. While randomization into the groups was initially planned, logistics of needing adequate time per session time available during college required adjustment. Participants were initially randomly allocated either to one of the intervention groups, and if unable to attend based on class schedules, re-allocated to the control group. This approach was adopted to meet logistics constraints which included the intervention time of about 1 h and allowing 5–10 min prior to intervention session and 20–30 min after the intervention. In addition, our previous recruitment strategies revealed that sessions before or after college classes will have limited study participants with potential dropout. Advantage of embedding the intervention during the college hours secured better recruitment and attendance. This strategy also allowed consistent use of limited access of meditation room in campus as well as availability of the lead instructor (UP), for each intervention. We used the same lead instructor throughout the study, but different, trained associates each semester for the assessment and data entry. Only the statisticians were blinded to the groups.

### Meditation Sessions

A trained instructor guided the students through a 25-min meditation three times per week for 8 weeks. During each session, participants sat in a simple cross-legged position and followed the instructor through a meditation process (Table 1). Each session was preceded by a 3-min relaxation period and concluded with a 2-min relaxation period, as well as 5 min for students’ questions. Each group was administered a pre-test at the beginning of the study and a post-test at or after the 8th week to assess affect at baseline and post-intervention.

**Table 1:** Interventions applied in each study group.

Session component	Sound-only (*mahapran*) *n* = 31	Color-focus only *n* = 34	Sound-color-focus *n* = 51	Control *n* = 26
11-min meditation	Sound-only during which subjects attempted to harmonize their sound with that of other participants.	Color-only imagery in which each participant focused on an imaginary green dot on their forehead.	Sound only	No meditation
3-min yellow color focus to maintain meditative state				
11-min meditation	Sound only	Color only	Color only	
Total meditation time	25 min	25 min	25 min	

Subjects in the intervention groups participated in meditation sessions of equal duration. Subjects in the control group did not participate in meditation sessions.

### Study Measures

#### Short-Term Working Memory

We assessed digital recall and recall processing; language recall and recall processing; and spatial recall and recall processing in all groups using the Alloway Working Memory Assessment short form (AWMA-2 SF; Alloway, 2007), a tool validated for individuals up to age 22. The digital recall test requires participants to hold an increasing number of digits in short-term memory and repeat the digits to the tester. The sentence recall test requires participants to listen to a sentence, remember the last word of each sentence, and then recite the last word to the tester. The spatial recall test requires individuals to mentally manipulate a series of shapes and to remember the sequence in which the shapes are placed. Spatial recall was available for control, color focus and sound and color focus groups only.

#### Affect

Affect was measured using the Positive and Negative Affect Schedule (PANAS). This consists of 2, 10-item self-report mood scales measuring the distinct dimensions of positive and negative affect (Watson et al., 1988). Using a scale from 1 (very slightly or not at all) to 5 (extremely), participants rated the extent to which they felt positive mood (e.g., enthusiastic, active, and alert) or negative mood (e.g., anger, disgust, and fear) throughout the previous week. The two scales, uncorrelated with each other, have shown high internal consistency and stability over time.

#### Selective Attention Capacity, Sustained Attention Capacity, and Impulsivity

These were measured using Connor’s Continuous Performance test 3rd edition (CPT-3™). This 14-min test consists of six groups of letters in which each letter of the alphabet is flashed on a computer screen individually in random order. Time between letters is variable, but averages 250 ms. Respondents are asked to press the spacebar after each letter except for letter X (which appears 36 times out of the 324 letters presented). Individual scores of variables measured indicate attention and impulsivity, and also allows derivation of Inattentiveness score from variables including detectability, omission and commission of errors as *t* scores as well as correct hit response time (Hit RT), and Impulsivity score from variables including Hit RT, commission of error and perseverations. This current version was used to assess the impact of mindfulness training in young adults with ADHD (Bueno et al., 2015).

#### Adherence to the Meditation Practice

This was assessed three times per session in all groups by measuring the duration of buzzing during *mahapran*. Participants were asked to take a deep breath, close their eyes, and emit a humming or buzzing noise for as long as they could with each breath.

#### Data Management and Statistical Analyses

Data were retrieved from CPT II software or directly entered into an Excel spreadsheet. The statistics team was blinded to the identity of the groups and participants and used IBM SPSS Statistics (Armonk, NY, United States) for data analysis. Generally, the data were presented as means and SDs (Essex et al., 2017) for continuous variables, while categorical variables were presented in percentages. Differences in means were used especially in pre- and post-treatment analyses. *t*-tests, Mann-Whitney *U* test (smaller sample sizes), and ANOVA were used to determine significant changes. Given number of variables, to avoid type 1 error, Bonferroni correction was applied and alpha < 0.0013 was the cut off for significance for the three intervention groups combined and controls. However, in comparing each variable between groups, sound focus, color focus, sound and color focus, and controls, for *a priori hypothesis* of interaction between methods of meditation, trends in values of *p* where variables had conventional alpha <0.05 were tabulated. For magnitude of effect changes from baseline were calculated using the confidence intervals from paired *t*-tests. Pearson’s correlation coefficient was used to assess the relationship between independent variables. Linear regression analysis was performed *post hoc* to assess group as dependent and change in measures from baseline as independent variables.

## Results

### Participants and Demographics

Following IRB approval, we allocated students (*n* = 178) at Florida International University (FIU) to one of three *preksha dhyana* intervention groups or to a control group: sound-only (*n* = 40), color-focus-only (*n* = 40), a combination of the two techniques, sound and color-focus (*n* = 60), or control group (*n* = 38; Table 2). The enrolled student population at FIU is 61% Hispanic or Latino, 13% black or African American, 15% white non-Hispanic, 4% Asian or Pacific Islander, and 7% other minority groups. About 58% of enrolled students are female and 42% male.

**Table 2:** Study participants.

	Control group	Meditation group		Total	
		Sound focus (*mahapran*)	Color focus	Combined techniques	
*N*	26	31	34	51	142
Mean Age (SE)	22.73 (3.99)	23.19 (6.56)	24.85 (8.40)	25.27 (6.78)	24.24 (0.76)
Male (%)	12 (46.2%)	12 (38.7%)	3 (8.8%)	13 (25.5%)	40 (28.2%)
Female (%)	14 (53.8%)	19 (61.3%)	31 (91.2%)	38 (74.5%)	102 (71.8%)

A total of 142 university students were allocated to either a control group or to one of three intervention groups.

**Figure 1:**
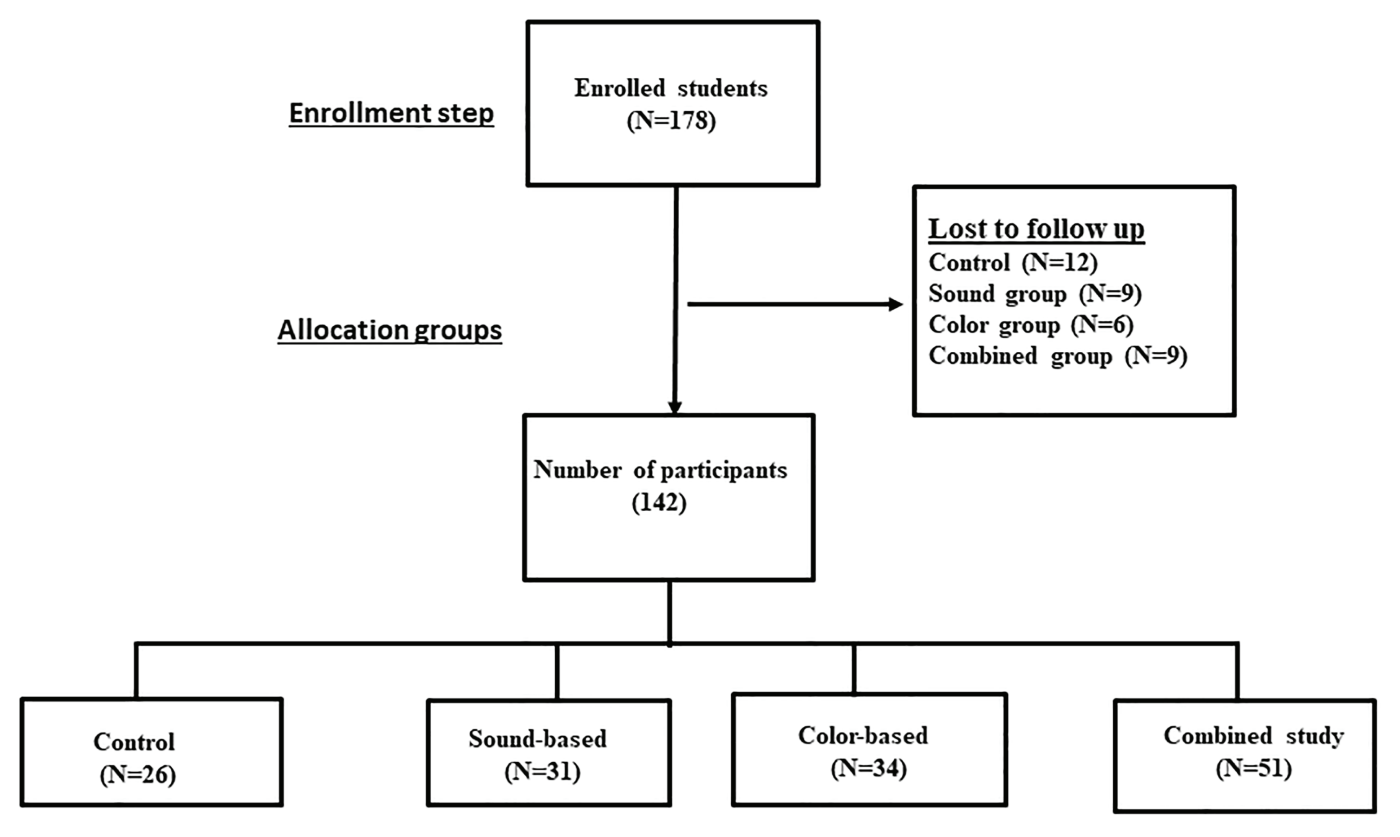
The flowchart of the study.

We used control group data from our previous study to estimate the sample size (*n* = 30) for a single intervention group (Pragya et al., 2014). Of enrolled participants, 142 completed the study. Thirty-six participants (20.2%) were lost to follow-up (12 controls, nine from the sound-only technique, six from color-focus technique, and nine from sound plus color-focus group). The flowchart of the study is illustrated in Figure 1. Overall, there were fewer males than females in all groups (*p* < 0.01), but all groups were equivalent in age (*p* = 0.32; Table 2).

### Assessment of *Preksha* Meditation

Overall, the three *preksha dhyana* groups combined (*N* = 116), after Bonferroni correction, did show average and maximum Buzzing time, Commission of Errors, Hit RT, Digital Recall, Listening Recall and Listening Recall Processing, Positivity, and Negativity improve significantly (*p* < 0.001). Using same stringency, none of the control group variables showed changes from baseline (Supplementary Table S1). In terms of magnitude of effect, changes in affect from baseline in the three *preksha dhyana* groups were calculated from the paired *t*-test confidence intervals. Negativity decreased by 19.8% (95% CI 9.0–30.6) and positivity increased by 10.4% (95% CI 2.7–18.1). This entire group also showed significant improvement from baseline in short-term working memory with digital recall increasing by 4.34 SD 11.8, CI 2.11–6.57, equivalent to 9.7% (95% CI 5.7–13.6) from baseline, and listening recall increasing by 8.79 SD 14.5 CI 6.1–11.5, equivalent to 11.1% (95% CI 6.2–15.9) from baseline. Spatial recall was assessed only in the color and sound and color focus groups and showed an increase of 4.41 SD 14.4 CI 1.0–7.9, equivalent to 5.7% (95% CI 1.1–10.3) from baseline (Supplementary Table S2).

To test whether differences existed between control group and the three *preksha dhyana* groups combined, independent sample *t*-test was run and Commission of errors *t* score (*F* = 0.316; *p* < 0.02), HitRT score (*F* = 1.904; *p* = 0.04), and Negative affect (*F* = 1.635; *p* = 0.04) showed trends but did not meet cut off or *p* < 0.0013 (Supplementary Table S3). In the three *preksha dhyana* groups combined, age was not correlated with differences in maximum duration of buzzing (*r* = 0.04, *p* = 0.72). In the control group, however, age showed a trend with the change in duration of buzzing (*r* = 0.45, *p* = 0.02; Supplementary Table S4).

In the three *Preksha* groups combined, the average buzzing time was correlated with differences in Omission T scores (*r* = 0.21, *p* = 0.03). Gender differences were seen in the control group, using the Mann-Whitney U test given sample size, for attention (improved Commission of error *t* score, *U* = 37.50; *p* = 0.03 in females) were noted. However, no gender differences were seen in the three *preksha dhyana* groups combined using *t*-test (Supplementary Table S5).

### Comparison of Individual Meditation Practices

**Figure 2:**
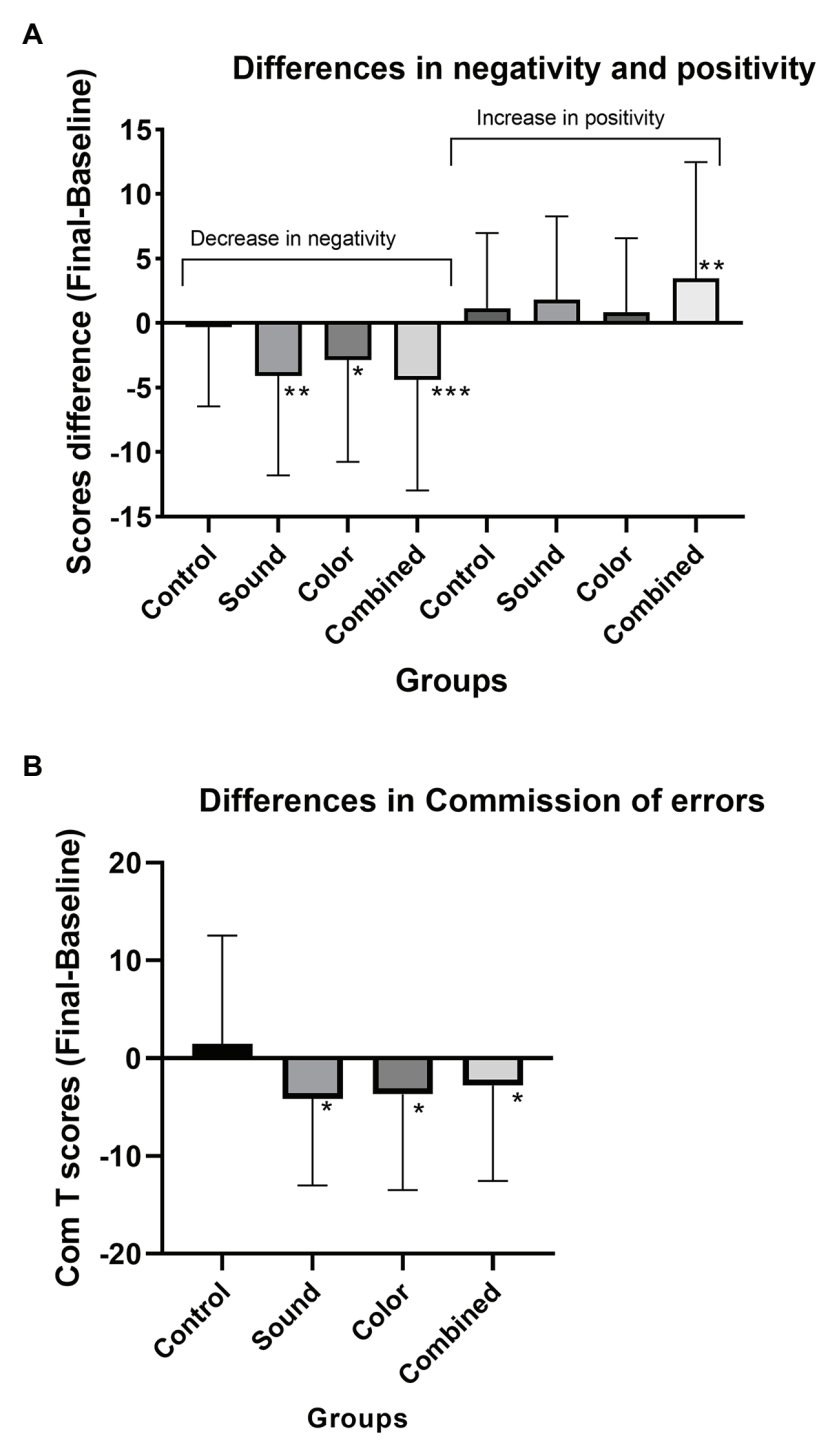
Differences in negativity and positivity from baseline **(A)** Commission of error differences from baseline **(B)** ^***^p < 0.01, ^**^p = 0.01, ^*^p < 0.05.

Results comparing the three meditation groups and controls using conventional alpha 0.05 as threshold to select variables for display are summarized in Table 3. In general values of *p* are lower in combined sound and color focus group than other groups. In addition, Spatial recall (*M* = -5.39; *SD* = 15.71; *p* = 0.02) and Spatial recall processing score (*M* = 6.06; *SD* = 14.71; *p* < 0.01) also showed trends of improvement in the combined sound and color-focus group, but not the color-focus only group nor control. Data of individual meditation practice are illustrated in Supplementary Tables S6–S8. Figure 2 depicts the differences in negativity, positivity, and commission of errors in every group (8 weeks postintervention compared to baseline).

**Table 3:** Paired *t*-test results.

	Control	Sound focus (*mahapran*)	Color focus	Combined
Change from baseline	*p*-value	*p*-value	*p*-value	*p*-value
Average buzzing time	0.74	0.01	0.08	0.01
Maximum buzzing time	0.57	0.03	0.01	0.05
Commission of error *t* score	0.53	0.03	0.02	0.03
Digital recall score	0.03	0.87	0.47	<0.01
Listening recall score	0.08	0.07	<0.01	<0.01
Listening recall processing Score	0.01	0.24	<0.01	<0.01
Positivity score	0.32	0.13	0.39	0.01
Negativity score	0.80	0.01	0.05	<0.01

Values of *p* changes from baseline are shown for the control, sound focus, color focus and combined techniques groups for the variables listed. Trend toward improvement in almost all variables in combined techniques group compared with the other groups is seen.

We used ANOVA to compare the difference in the change in score from baseline to post- intervention across groups (control group and the each of the three meditation groups). At least one group was significantly different for HitRT and Digital Recall (Supplementary Table S9). Since each group varied in number of participants, we applied the Bonferroni procedure to determine whether statistically significant differences existed between the groups. Change in digital recall from pre- to post- meditation differed significantly between the combined techniques and sound-only (*mahapran*) intervention groups (*F* = 5.618; *p* < 0.01,) and between the combined techniques and color-focus-only intervention groups (*F* = 7.307; *p* = 0.02), but did not differ significantly with the control group (*F* = 5.490; *p* = 0.24). Interestingly, change in maximum buzzing duration was significantly positively correlated with a change in listening recall (*r* = 0.53, *p* = 0.01) and change in average buzzing duration was significantly positively correlated with the change in attention (Commission of Error *t* score, *r* = 0.21, *p* = 0.03). In terms of semesters, no specific differences were seen that were not accounted for by changes in groups, especially the control group (Supplementary Table S10). A multiple regression was calculated to predict group based on variables. A significant regression equation was found [*F*(4,130) = 4.018, *p* < 0.004], with an R^2^ 0.11. The predicted group is equal to 1.501–0.028 (Delta Negativity) – 0.01 (Delta Commission of Errors) + 0.021 (Delta Digital recall) + 0.008 (Delta Listening Recall), where Delta variable is differences from baseline in respective scores. Group allocation increased by 1.501–0.028 in Negativity, −0.01 in Commission of errors, + 0.08 in increase in Listing Recall, + 0.021 in Digital Recall. Thus, Group allocation increased in increments of + 0.021 in Digital Recall, 0.008 Learning Recall and decrements of 0.028 in Negativity, and 0.01 in Commission of Error. By adding Gender as a variable, the regression equation was *F*(5,129) = 3.885, *p* < 0.003 with R^2^ improved to 0.13. The variables Negativity, Commission of Errors, Digital Recall and Learning Recall as well as gender were significant predictors accounting for 13% of responses to intervention.

## Discussion

Combined *Preksha dhyana* meditation groups demonstrated significant improvements in affect, attention, and short-term memory in a prospective cohort-controlled study. Improvements over baseline in affect for both Positivity and Negativity, as well as inattention and impulsivity, and improved short-term memory for numbers and words were seen. Benefits were seen in both genders. Having shown efficacy of *preksha dhyana* we focused on the primary goal of this study, which, was to evaluate the impact of individual techniques used in the *preksha dhyana* meditation practice with the long term goal of harnessing individual meditation techniques in isolation or varied combinations to personalize therapy for specific disorders, such as ADHD.

In terms of trends within the three *preksha dhyana* meditation groups, study participants showed some improvement over baseline for affect, short-term memory, and attention. The degree of improvement varied between the sound-only and color-focus only meditation techniques and was larger and more widespread when both techniques were combined. Participants using the sound technique (*mahapran*) showed cognitive performance improvement on CPT-3™ with reduced commission of errors, also a marker of attention. Students in this group also exhibited decreased negative affect from baseline. We previously reported the benefits of this technique on students with learning disabilities and noted the ease with which the technique was learned by this group (Pragya et al., 2014).

Participants using the color-focus technique alone also showed improved scores for (commission of errors, also a marker of attention) and affect (decreased negativity). Short-term working memory improved significantly from baseline (listening recall and listening recall processing). In participants who used both meditation techniques (combined sound and color focus), short term memory for digital recall, spatial recall, and spatial recall processing improved from baseline. Affect improved significantly from baseline (both decreased negativity and increased positivity), an outcome not achieved when using either technique individually. This suggests that both techniques are associated with beneficial outcomes and suggest these benefits to be incremental when combined, despite same total time of meditating.

In terms of magnitude of effect changes in affect in the study group using both techniques were substantial, with negativity decreasing (mean 19.8%) and positivity improving (mean 10.4%). This group also showed significant improvement from baseline in short-term working memory with digital recall (mean 9.7%), listening recall (mean 11.1%), and spatial recall (mean 5.7%). Similar changes were shown for attention, with decreased commission of errors. The buzzing duration was associated with increased attention (Commission of Errors *t* score). Using multiple regression, five variables accounted for 13% of the improvement seen. These were Negativity reduction, Commission of Error reduction, and an increase in digital memory and learning memory. Gender as female was also associated with response. This further underscores incremental benefit from single intervention to combined intervention.

In a similar sample size, mindfulness, a comparable form of meditation with roots from ancient Buddhism and Yoga, revealed measurable effects in 156 college students (Hirshberg et al., 2018). The participants were novices to mindfulness, and they were allocated to one of four brief practices, breath awareness, loving-kindness, gratitude, or to an attention control condition. The study revealed that gratitude training significantly improved positive affect compared to breath awareness, while loving-kindness led to significantly greater reductions in implicit negative affect compared to the control condition immediately after brief practice. The study did not seek to assess cumulative impact of these techniques on the same population. In other studies, mindfulness showed improvement of mood in college students, especially depression as well as stress reduction (Mendes and Palmer, 2016; Sundquist et al., 2017; Alzahrani et al., 2020).

The prefrontal cortex has been associated with enhanced cognitive focus and the parietal lobe with improved spatial cognition (Somers and Sheremata, 2013). Our results support these data: the study group using a focused meditation technique (in this case, color) showed increased attentiveness and spatial recall ability, while this was not the pattern seen in the sound-based group. In a small but randomized controlled trial, short-term training with meditation over 8 weeks also lead to increased functional connectivity between the amygdala and a region implicated in emotion regulation [ventromedial prefrontal cortex (VMPFC); Kral et al., 2018]. This finding suggests neuroplasticity even in short term intervention.

Other studies showed that different techniques activate different brain areas and stimulate different brain wave patterns. In experienced meditators, EEG (Dunn et al., 1999; Lehmann et al., 2001) and functional magnetic resonance imaging (fMRI; Wang et al., 2011) have shown that different meditative techniques correlate with variable activation in different brain areas. Wang et al. (2011) showed that focused meditation practice affected subjects’ prefrontal cortex, while a breathing-based practice affected the limbic system and other areas associated with mood or affect (Wang et al., 2011). Taken together, these studies showed how different techniques, added together, can lead to a cumulative effect. However, further work is warranted to understand the affected physical regions in the brain, but also the cellular level mechanisms that may be involved in such responses even in the short term as we are reporting.

The study strengths included the large group size, subject retention in the intervention group, and using the length of buzzing as in internal marker of adherence. In addition, the use of Bonferroni correction reduced type 1 error. Study limitations include the inability to fully randomize groups due to students’ schedule constraints, and availability of appropriate space, although we do not believe this added significant systematic bias. The gender differences in the groups may also introduce a bias in the study. In addition, the attrition rate of the control group was larger than expected and reduced the effective power of the study for cross-group comparison. Ideally, the control group would have been exposed to a sham or active intervention with a similar program pattern; however, to date, such control groups have not been universally standardized or validated for studies on meditation.

## Conclusion

*Preksha dhyana* is a multi-step Jain meditation process using two specific techniques: *mahapran* (a sound and breathing technique) and a color-focused imagery technique. This study showed incremental as well as differential benefits from both techniques, with the greatest benefits when both techniques are combined. The magnitude of beneficial effects on affect and on short-term memory was statistically significant, even with a brief intervention. Further study to assess both cumulative and differential effects of other *preksha dhyana* techniques specifically and all meditative practices in general can contribute to individualized meditation modalities to treat disorders such as attention deficit hyperactivity disorder.

## Data Availability Statement

The raw data supporting the conclusions of this article will be made available by the authors, without undue reservation.

## Ethics Statement

The studies involving human participants were reviewed and approved by Florida International University (FIU; IRB-17-0108-CR02). The patients/participants provided their written informed consent to participate in this study.

## Author Contributions

SUP and NM contributed equally to this work. Psychological tests were applied by PU and NM. Additionally, SM, MJ-F, and SG were involved in the statistical analyses. BA, NM, PV, SM, SCP, and DM were involved in data collection, data management, and writing the manuscript. All authors contributed to the article and approved the submitted version.

### Conflict of Interest

The authors declare that the research was conducted in the absence of any commercial or financial relationships that could be construed as a potential conflict of interest.

## Supplementary Material

Supplementary Table S1 The control group (*n* = 26) assessment data. Significant differences for digital recall score (mean = −3.33, *p*-value = 0.03), listening recall processing score (mean = 9.36, *p*-value = 0.01) were observed. Values of *p* did not meet Bonferroni criteria.

Supplementary Table S2 Assessment of different variables among all the meditation groups (*n* = 116). Values of *p* are shown for some of the variables including those with uncorrected *p* < 0.05. ^*^indicates significance after Bonferroni correction.

Supplementary Table S3 Analysis between and within groups. At least, one group showed statistical differences (*p* < 0.05) in HitRt score and digital recall score.

Supplementary Table S4 Correlations between age and improvement.

Supplementary Table S5 Significant differences in improvement between genders for meditation group.

Supplementary Table S6 The sound focus (*Mahapran*) meditation (*n* = 31). (*p* < 0.05 was considered significant).

Supplementary Table S7 The color focus meditation (*n* = 34). (*p* < 0.05 was considered significant).

Supplementary Table S8 Assessment of the combined group meditation (*n* = 51). (*p* < 0.05 was considered significant).

Supplementary Table S9 *Post hoc* test (Bonferroni test was used to account for sample size differences) for the three statistically significant variables. *Denotes statistically significant mean value.

Supplementary Table S10 Significant differences in improvement between semesters for control group.
